# Current Perspectives on Uniparental Mitochondrial Inheritance in *Cryptococcus neoformans*

**DOI:** 10.3390/pathogens9090743

**Published:** 2020-09-10

**Authors:** Amber R. Matha, Xiaorong Lin

**Affiliations:** Department of Microbiology, University of Georgia, Athens, GA 30602, USA; Amber.Matha1@uga.edu

**Keywords:** *Cryptococcus neoformans*, uniparental mitochondrial inheritance, mating, bisexual reproduction, pheromone sensing pathway

## Abstract

The mitochondrion is a vital organelle in most eukaryotic cells. It contains its own DNA which differs from nuclear DNA, since it is often inherited from only one parent during sexual reproduction. In anisogamous mammals, this is largely due to the fact that the oocyte has over 1000 times more copies of mitochondrial DNA than the sperm. However, in the isogamous fungus *Cryptococcus neoformans*, uniparental mitochondrial inheritance (UMI) still occurs during sexual reproduction. It is proposed that UMI might have evolved in the last common ancestor of eukaryotes. Thus, understanding the fundamental process of UMI in lower eukaryotes may give insights into how the process might have evolved in eukaryotic ancestors. In this review, we discuss the current knowledge regarding the cellular features as well as the molecular underpinnings of UMI in *Cryptococcus* during the mating process, and open questions that need to be answered to solve the mystery of UMI in this eukaryotic microbe.

## 1. Introduction

Mitochondria, a characteristic organelle of eukaryotes, are thought to have evolved through an endosymbiotic relationship between an ancient archaeal cell and an alpha-proteobacterium [1]. This symbiotic relationship is likely to have evolved in the last common ancestor of *Eukaryota* [2,3]. In addition to being the powerhouse of the cell, the mitochondrion is responsible for diverse critical cellular events such as aerobic respiration, apoptosis, and calcium signaling [1]. Likely due to its endosymbiotic origin, the organelle contains its own DNA (mtDNA), which differs from the host nuclear DNA in a variety of ways. First, the mitochondrial genome is a fraction of the size of the nuclear genome: in humans, mtDNA consists of a circular DNA molecule containing approximately 16,000 base pairs, compared to the 3 × 10^9^ base pairs of the nuclear genome arranged in linear chromosomes. The small mitochondrial genome only codes for ~13 proteins. Consequently, most mitochondrial resident proteins are derived from nuclear coding genes. Second, each mitochondrion can contain many copies of its genome, whereas the nucleus only contains one or two copies of its genome for a haploid or a diploid cell [4]. Third, mitochondria replicate and undergo fission and fusion independently of the cell cycle, which is dictated by nuclear division.

One of the most interesting features of mitochondria is the organelles’ inheritance during sexual reproduction. Evidence that Mendel’s laws of inheritance does not apply to organelle DNA was first shown in 1909 in independent studies by Carl Correns and Earl Bauer [5]. They found that chloroplast plastids in different plant species were inherited maternally (Corren) or biparentally (Baur) [6]. Similarly, mtDNA of meiotic progeny is inherited from only one parent in many organisms. A classic example is maternal mitochondrial inheritance in humans, discovered in 1980 [7]. Here, the cellular features of the parental cells greatly impact the mtDNA inheritance pattern: the sperm, excluding the tail, is 10–15 μm in length and has only 50–75 copies of mtDNA. By contrast, the egg is 100 μm in diameter and has over 100,000 copies of mtDNA [8,9,10]. Although the sperm mitochondria are able to enter the egg in most mammals [11], the sharp disproportion of the number of mitochondria in the egg and sperm is one decisive factor contributing to UMI [11]. Another feature contributing to UMI is that the egg is stationary while the sperm is actively moving to achieve cell fusion. The high energy demand of the sperm while en route to fertilization increases the generation of reactive oxygen species [12]. This could damage the paternal mtDNA, which is then recognized by the zygote, destroyed, and eliminated from the progeny [13]. 

Although the drastic difference in mtDNA copies can largely explain UMI in mammals, this cannot apply to UMI during sexual reproduction in isogamic lower eukaryotes like the green alga *Chlamydomonas reinhardtii* and the fungus *Cryptococcus neoformans* where both parental cells exhibit similar size and morphology. The ancient ancestor of eukaryotes engaging in bisexual reproduction was likely to have been isogamic [14], and thus investigation of the mechanisms underpinning UMI in modern eukaryotic microbes provides a unique vantage point to understand the evolution of UMI. 

The genetic amenability and resources/tools developed for *C. neoformans* has rendered it a model organism in the study of UMI. Collectively with studies of other organisms, mitochondrial inheritance studies of *Cryptococcus* offer a platform for comparative analyses of UMI in eukaryotes and its potential evolutionary origin. Here, we summarize the past investigations that have documented the mating process and the associated cellular features and molecular factors contributing to UMI in *C. neoformans*. We discuss controversies and challenges that need to be addressed to resolve the mechanism underpinning UMI in this eukaryotic microbe. 

## 2. Discovery of UMI in Fungi and *Cryptococcus*

Studies of mitochondrial inheritance in eukaryotic microorganisms were initiated in the 1940s when petite mutations were discovered in *Saccharomyces cerevisiae* [15]. A petite mutation slows growth, yielding small or “petite” colonies. In a bisexual cross between a wildtype and a petite cell, the petite cell phenotype was lost completely in haploid F1 progeny, indicative of non-Mendelian inheritance [15]. When these progenies were allowed to replicate mitotically, petite colonies could re-emerge. It was discovered that the petite phenotype was due to lack of mtDNA and these cells are incapable of aerobic respiration [15]. A portion of the F1 progeny contained mitochondria from both parents (ones with mtDNA and ones without), which allowed for the re-emergence of the petite phenotype in the mitotic daughters of the F1 cells. This and other studies in *S. cerevisiae* also indicate that strict UMI is not typically present in this model yeast. During mating, two *S. cerevisiae* yeast cells of opposite mating types (**a** and α) fuse and form a zygote with a conjugation tube connecting the parental cell bodies. Meiotic daughter cells inherit mtDNA from both parents [16,17], but the proportions of each parental mtDNA depends on the position from which the daughter cell emerges from the zygote: buds that originate from the middle of the zygote are heteroplasmic and contain both parental mtDNA, and buds that originate from either end of the zygote are nearly homoplasmic [18]. These heteroplasmic daughter cells can obtain homoplasmy within 20 mitotic divisions [19].

UMI in *C. neoformans* was discovered in 2000 [20]. The tester strain JEC20**a** of serotype D was mated with different α clinical isolates of serotype A. The differences in the mtDNA sequences of serotype A and serotype D allowed for the determination of the origin of mtDNA in the progeny via PCR and Restriction Fragment Length Polymorphism (RFLP) analysis. In all crosses, only mtDNA derived from JEC20**a** were recovered from basidiospores (Table 1) irrespective of the radial distance of the sampled basidiospores from the center of the mating mixture. As cryptococcal mating is a heterogeneous and sporadic process, such distance may not accurately reflect the elapsed time (or the number of divisions) between cell fusion and sporulation. Nonetheless, UMI is observed within 24 h of mating in the cell fusion products prior to the generation of abundant mating hyphae [21], suggesting that UMI is established soon after cell fusion. That said, the precise stage at which UMI is established is yet to be determined.

## 3. Cellular Features during Mating and Uniparental Mitochondrial Inheritance in *C. neoformans*

**Figure 1:**
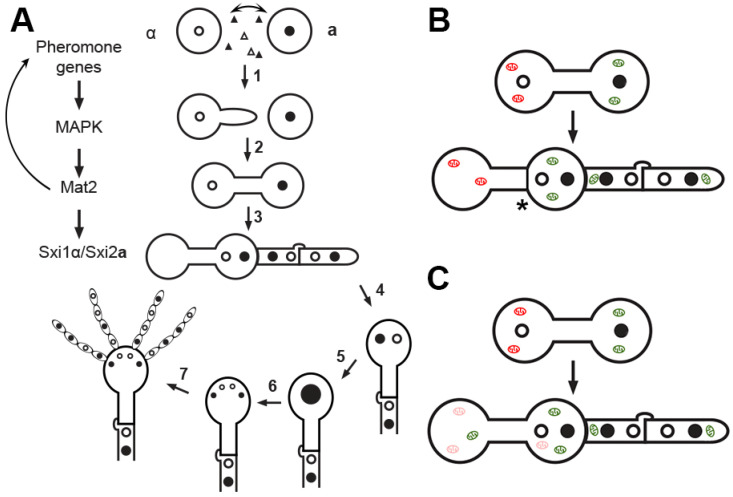
*Cryptococcus* bisexual reproduction. (**A**). 1. Two parental cells of opposite mating types (**a** and α) are in close proximity. The **a** cell secretes pheromone first (filled triangles) and the α cell responds by secreting α pheromone (open triangles) and sending a conjugation tube. Pheromone sensing and response pathway is signaled through the Cpk1 MAP kinase cascade, with the ultimate transcription factor Mat2 controlling gene expression. 2. Mat2 dictates conjugation tube formation, cell fusion, and the formation of the zygote. 3. Upon cell fusion, Sxi1α/Sxi2**a** form a complex, which directs the formation of dikaryotic hyphae where two parental nuclei congress but do not fuse. 4. The tip of the dikaryotic hypha swells to form the basidial head. 5. The two parental nuclei fuse in the basidial head. 6. Meiosis occurs. 7. Repeated mitosis and sporulation occur. Spores germinate into yeasts and the process can repeat. (**B**). One hypothesis for UMI in *Cryptococcus neoformans* posits that spatial segregation of α mitochondria inhibit their entering into the mating hyphae. (**C**). Another hypothesis suggests that α mitochondria are actively degraded upon formation of the zygote via a presently unknown mechanism.

As cellular features during bisexual reproduction could have a great impact on UMI in *C. neoformans*, it is critical to define both pre-zygotic mating events and the post-zygotic development. Mating in *C. neoformans* is controlled by the mating type locus, which can encode either the **a** or the α allele. Bisexual mating is stimulated by nutrient deprivation and the presence of a compatible mating partner in close proximity. In the currently accepted model, α cells produce a conjugation tube in response to pheromone exuded by the nearby **a** cells (Figure 1A). This conjugation tube fuses to the **a** cell, forming the dumbbell shaped zygote. The nucleus from one parent travels through the conjugation tube and a dikaryotic hypha forms from one of the poles of the zygote. The dikaryotic hypha can extend at the tip indefinitely before generating a basidium head where two parental nuclei fuse. This is then followed by meiosis and sporulation (Figure 1A). Previous work has shown that the α nucleus traverses the conjugation tube after zygote formation [22,23] and the mating hypha originates from the side of the original **a** cell of the zygote [22] (Figure 1A). In the study by McClelland et al. [22], the two parental cells were labeled with different AlexaFluor dyes prior to mating. In the zygote, two nuclei were observed as expected based on DAPI staining: one in the **a** cell (labeled red) and the other in the conjugation tube. This observation led to the conclusion that the α nucleus leaves the parental cell and travels through the conjugation tube to join the **a** nucleus in the parental **a** cell. Recently, Nishimura et al. [24] reported that the **a** nucleus is located inside of the conjugation tube. However, the images presented in Nishimura’s report do not allow unequivocal distinction of the assumed “zygote” from other neighboring cells. The challenge to the generally accepted findings from McClelland et al. has yet to be independently verified.

The uniparental inheritance of **a** mitochondria could be explained by an uneven mix of the cytoplasm and consequently the mitochondria after cell fusion. This could prevent α mitochondria from entering the mating hyphae, which eventually develop into fruiting bodies including basidiospores (Figure 1A). Evidence supporting such cellular features, like the origin of the conjugation tube and the mating hyphae depicted in Figure 1A, comes from a few studies. In one study discussed earlier [22], the differentially labeled parental cells by AlexaFluor dyes allowed the authors to determine which parent sent the conjugation tube and which side of the zygote the mating hypha originated from. The finding that α cells send conjugation tubes in these earlier studies [22,25] was later verified by Sun et al., who showed that conjugation tubes emerge only from α cells that are fluorescently labeled with the cytoplasmic protein Cna1 [23]. Upon the formation of the mating hypha, the Cna1 fluorescent signal was distributed throughout the whole zygote. This observation suggests that uneven cytoplasmic mixing is unlikely to be the driving force of UMI. Contrary to the aforementioned model, Nishimura et al. reported that a conjugation tube can originate from the **a** cell [24]. Thus, it remains to be established if the α nucleus migrates through the conjugation tube to meet the **a** nucleus on the other side of the zygote and if migration of organelles, including nuclei and mitochondria, follows the same dynamics of cytoplasm mixing.

The distribution of the parental mitochondria within the zygote is also a topic of controversy. As mentioned previously, Sun et al. showed that the α mitochondria extend into the conjugation tube based on the labelling of nuclear encoded mitochondrial protein Hem15. Nishimura et al. reported an opposite observation based on the labelling of nuclear encoded mitochondrial protein Atp2 [24]. In this latter study, in a cross between an α strain labeled with Atp2-mCherry and a nonlabelled **a** strain, the mCherry labeled mitochondria were specifically restricted to the α parental cell and did not extend into the conjugation tube. Instead, it appears that unlabeled **a** mitochondria were inside the conjugation tube based on the Rhodamine 123 staining of mitochondria. It is important to note that any nuclear encoded mitochondrial protein (e.g., Atp2 or Hem15) will be first translated in the cytosol and then shuttled to mitochondria. When the zygote forms, the cytoplasm of the mating cells mixes. Thus, the newly synthesized mitochondrial proteins, regardless of which parental nuclear DNA carries the gene, should be able to translocate to mitochondria derived from either the **a** or the α parent. Therefore, imaging cryptococcal cells engaged in mating before and after cell fusion, either through time-lapse live imaging or carefully monitoring the developing mating partners and the forming zygote, is critical to avoid this complication.

There is some evidence suggesting that mitochondrial mixing may differ from cytoplasmic mixing in the zygote (Figure 1B). According to Sun et al., mitochondria from the α cell are present within the conjugation tube, but there is a spatial separation between each parent’s mitochondria once the zygote forms, based on microscopical observation of a dark region between GFP labeled mitochondrial protein Hem15 from both parental cells [23]. Future studies are needed to confirm that the mitochondrial populations on either side of the separation are truly distinct and to confirm the nature of the separation if it truly exists. It would be fascinating to know if this separation of mitochondrial populations coordinates with nuclear migration in a manner that allows the two parental nuclei to migrate to the next phase of zygote growth while inhibiting the participation of α mitochondria.

Separation between mitochondrial populations has been observed during mitosis in *S. cerevisiae* and meiosis in *Schizosaccharomyces pombe* [26,27]. The daughter cells of a heteroplasmic mother cell can obtain a homoplasmic state due to the tethering of some mitochondria to the mother’s cell membrane through the mitochondria–ER cortex anchor (MECA) while a subset of the mitochondria are transported to the bud on actin [26]. In *S. cerevisiae,* MECA is composed of two proteins, Num1 and Mdm36 [26]. Mdm36 facilitates the assembly of the anchor protein Num1 into MECA by forming a bridge between Num1 proteins. During sexual reproduction in *S. pombe*, the homologue to Num1 (Mcp5) restricts the mixing of the mitochondria by tethering the mitochondria populations to the poles of the zygote after cell fusion and nuclear fusion [27]. Following meiosis, the two pairs of ascospores inherit the mtDNA nearest to them, allowing for homoplasmic daughter cells. There is a homolog of *NUM1* but not *MDM36* in *C. neoformans*. Nonetheless, a similar mechanism could be taking place in restricting the mixing or the movement of mitochondria during mating.

It is clear that more investigations into the cellular features and organelle distribution dynamics during bisexual mating are warranted, using time-lapse imaging or still imaging at multiple time points to catch each differentiation stage if necessary. These features could have a great impact on the strict control of UMI. Consistent with this idea, environmental factors that perturb the mating process also perturb UMI. For instance, laboratory crosses of *C. neoformans* are typically performed at room temperature in the dark [25]. High temperatures and UV irradiation increase the leakage of UMI and more progeny inherit *MAT*α mtDNA [28] (Table 1). Thus, understanding the cellular and molecular features of the mating process holds the key to our understanding of UMI. 

## 4. Factors Important for Uniparental Mitochondrial Inheritance in *C. neoformans*

Both prezygotic differentiation and postzygotic development are governed by signaling pathways. It is postulated that disruption of regulators of the mating process would then alter UMI. Of the signaling pathways that control the early stages of mating, the pheromone sensing and response Cpk1 MAPK pathway plays the most prominent role (Figure 1A). The impact of mutations of some of the components of this pathway on the mitochondrial inheritance pattern has been examined and a summary of these findings can be found in Table 1. Mat2, the transcription factor that regulates the pheromone response pathway, is highly induced during the prezygotic phase of mating before the parental cells fuse. Interestingly, Mat2 determines which parental mitochondria persist in the progeny: overexpression of *MAT2* in the α parent results in the dominant inheritance of the α mitochondria and vice versa (Table 1). Although Mat2 is not encoded by the mating type locus, this transcription factor controls the expression of a large number of genes involved in the pheromone pathway, including those encoded by the mating type locus such as pheromone genes *MF*α/**a**, pheromone receptors *STE3*α/**a**, and transcription factors *STE12α*/**a** [30]. Overexpression of Mat2 could alter cellular features or organelle distribution during mating, or it could alter processes involved in the maintenance or degradation of mitochondria. The downstream targets of Mat2 and their roles in UMI have yet to be investigated.

Recently it was shown that Crg1, a negative regulator of the pheromone pathway, plays a modest role in UMI [23]. Crg1 deletion enhances the pheromone sensing and response pathway. When both parents are *crg1*Δ mutants (bilateral cross), leakage of progeny inheriting α mitochondria increases, although there is still a strong preference for **a** mitochondria (Table 1). Conjugation tubes sent from both parents were observed when *crg1*Δ mutants mated [23], which might account for some α mitochondrial inheritance. How Crg1 exerts its impact on UMI is unknown, but it is likely to be through its role as a negative regulator of the Cpk1 pheromone pathway. Mat2, the UMI decisive regulator, controls the expression of *CRG1.* The transcript level of *CRG1* in *mat2*Δ is ~10-fold lower than that of the wild type based on our RNA-seq data. Overall, the pheromone response pathway regulates cellular differentiation and has a huge impact on the pattern of UMI.

Another set of transcription factors that play an important role in UMI are the homeodomain proteins Sxi1α and Sxi2**a**. These are the cell identity factors encoded by cryptococcal mating type locus (α cells carry *SXI1*α and **a** cells *SXI2***a**). In *S. cerevisiae*, the cell identity factors 1α and 2**a** from α and **a** cells form a complex in the zygote. The heterocomplex represses genes related to haploid cell function while promoting transcription of genes involved in diploidization, meiosis, and sporulation [31]. In *C. neoformans*, Sxi1α and Sxi2**a** behave similarly after they form a complex in the zygote [32], and the complex is required for the formation of the mating hyphae and other postzygotic development during bisexual reproduction [33,34]. In accordance with their cell identity function, when *SXI2**a*** is artificially expressed in an α cell or *SXI1α* in an **a** cell, the cell with both identity factors alone undergoes development typical of post-zygotic bisexual development [33,34]. When either *SXI1α* or *SXI2**a*** is deleted from one parent, cell fusion/mating can still occur. However, the zygotes fail to generate mating hyphae and they inherit mitochondria biparentally [29] (Table 1). Interestingly, if Mat2 is overexpressed in the **a** parental strain, uniparental inheritance of **a** mitochondria is largely restored even in the absence of a functional Sxi1α/Sxi2**a** complex [29] (Table 1). Thus, there appears to be an intimate relationship between Mat2 and the Sxi1α/Sxi2**a** complex. Not surprisingly, *SXI1*α and *SXI2***a** are direct targets of Mat2 [35]. The gene targets of the Sxi1α/Sxi2**a** complex have been identified [32], but how the Sxi1α/Sxi2**a** complex and their targets help control UMI remains unknown.

How do these genes control UMI? One possibility is that Mat2 and/or the Sxi1α/Sxi2**a** complex regulate genes involved in the degradation of the mitochondria via mitophagy. The nematode *Caenorhabditis elegans* relies on autophagy to eliminate the sperm’s mitochondria from the fertilized egg [11]. In some mammals, the mitochondria in the sperm are ubiquitinated and degraded by the lysosome and/or proteasomes [11]. However, autophagy does not seem to play any important role in UMI in lower eukaryotes tested so far. For example, deletion of *ATG11*, a receptor mediated mitophagy protein, does not affect UMI in *Ustilago maydis* [36,37], another basidiomycete that switches to dikaryotic hyphal growth after cell fusion of compatible mating partners. Autophagy does not play a role in mitochondrial inheritance in *C. neoformans* either. Nishimura et al. have shown that disruption of *ATG8* (responsible for the creation of the autophagosome vesicle membrane [38] and *NUC1* (an endonuclease involved in mtDNA degradation) do not have an effect on the preferential inheritance of *MAT***a** mtDNA inheritance in *Cryptococcus* [24] (Table 1). Consistently, we found that a unilateral cross of wild type with either *atg3*Δ or *aif1*Δ mutant defective in autophagy-related E2-like conjugation enzyme and mitochondrial nuclease respectively yielded the same UMI pattern as a cross between two wild type partners.

Methylation and ubiquitination are common organelle degradation pathways in *C. reinhardtii* and in mammals, respectively [11]. In the haploid alga *C. reinhardtii*, chloroplast DNA (cpDNA) is inherited from the mt^+^ parent and the cpDNA from the mt^-^ parent is degraded soon after zygote formation [39]. Treating cells with the methylation inhibitor 5-aza-22′-deoxycytidine (5-adc) disrupts chloroplast inheritance in *C. reinhardtii*. However, 5-adc treatment has no effect on UMI in *C. neoformans* [28]. In mammalian cells, sperm mitochondria are ubiquitinated during spermatogenesis before the fertilization event occurs [40]. Ubiquitination marks paternal mitochondria for destruction. Inhibition of ubiquitination by ammonium chloride therefore disrupts UMI in mammalian cells [41]. However, treating *C. neoformans* with ammonium chloride failed to alter UMI [28]. Some uncharacterized mechanisms for selective preservation of the **a** mitochondria or degradation of the α mitochondria must operate in *C. neoformans* (Figure 1C).

## 5. Conclusions

The exact mechanism for uniparental inheritance of mitochondria in *C. neoformans* remains unknown for now. However, there is mounting evidence that the inheritance pattern of mtDNA is intimately related with the cellular differentiation and the genes involved in the mating process. Controversy exists in this field relating to which parent develops a conjugation tube during mating, and whether the mitochondria from the α parent is able to enter the conjugation tube and subsequently the mating hypha and spores. So far, some key UMI regulators such as the prezygotic transcription factor Mat2 and post-zygotic complex Sxi1α/Sxi2**a** have been identified. However, the structural components of the downstream factors that execute their regulation in UMI remain unknown. Such interesting scientific questions must be answered to resolve these disputes and to elucidate the fundamental process of UMI in this eukaryotic microbe.

## Figures and Tables

**Table 1:** Summary of mitochondria inheritance patterns.

Cross Description	Cell Type Tested	mtDNA Inheritance %, a/α	Reference
Aα × D**a**	Basidiospores	Uniparental from *MAT**a*** **100**/0, n = 446	[20]
Aα × D**a**	Diploid zygote	Uniparental from *MAT**a*** **82**/4, n = 50	[29]
Aα *MAT2^OE^* × D**a**	Diploid zygote	Biparental (mostly from MATα) **35**/60, n = 87	[29]
Dα × A**a** *sxi2**a***Δ	Diploid zygote	Biparental (mostly from MATα) **24.5**/69.4, n = 49	[29]
Dα *sxi1α*Δ × A**a**	Diploid zygote	Biparental (mostly from MATα) **25**/75, n = 48	[29]
Dα *sxi1α*Δ × A**a** *MAT2^OE^*	Diploid zygote	Uniparental from *MAT**a*** **73.8**/23.8, n = 42	[29]
D**a** (mtA) *atg8*Δ × Dα (mtD) *atg8*Δ	Diploid zygote	Uniparental from *MAT**a*** **92.3**/7.7, n = 52	[24]
D**a** (mtA) *nuc1*Δ × Dα (mtD) *nuc1*Δ	Diploid zygote	Uniparental from *MAT**a*** **95.7**/4.3, n = 47	[24]
D**a** (mtA) × Dα (mtD) UV radiation	Diploid zygote	Biparental **54**/40, n = 163	[28]
D**a** (mtA) × Dα (mtD) High temperature (33 °C)	Diploid zygote	Biparental **53**/40, n = 184	[28]
Aα *crg1*Δ × A**a** *crg1*Δ	Basidiospores	Biparental (mostly from *MAT**a***) **80**/20, n = 40	[23]

Note: Serotypes (capital letter A or D) are defined by immunoreaction patterns against capsule. Serotypes correlate with molecular types that are classified based on DNA sequence polymorphism. The DNA sequence polymorphisms of mtDNA between these serotypes allow their distinction by PCR or RFLP (Restriction Fragment Length Polymorphism). Biparental inheritance refers to an inheritance pattern where the mtDNA of both parents are inherited. All crosses involved α-**a** bisexual mating between two haploid parental strains. Due to the recovery of some recombinant mitochondria or the inheritance of both parental mitochondria in one progeny, the percentage of some of the crosses does not total 100%.
